# Determination of Gait Events and Temporal Gait Parameters for Persons with a Knee–Ankle–Foot Orthosis

**DOI:** 10.3390/s24030964

**Published:** 2024-02-01

**Authors:** Sumin Yang, Bummo Koo, Seunghee Lee, Dae-Jin Jang, Hyunjun Shin, Hyuk-Jae Choi, Youngho Kim

**Affiliations:** 1 Department of Biomedical Engineering, Yonsei University, Wonju 26493, Republic of Korea; abbey0909@yonsei.ac.kr (S.Y.); beommo@yonsei.ac.kr (B.K.); fhrm502@yonsei.ac.kr (S.L.); 2 Korea Orthopedics and Rehabilitation Engineering Center, Incheon 21417, Republic of Korea; rehajang@kcomwel.or.kr (D.-J.J.); hjshin@kcomwel.or.kr (H.S.); choi4215@kcomwel.or.kr (H.-J.C.)

**Keywords:** gait event detection, knee–ankle–foot orthoses (KAFO), gait parameters, IMU sensor, thigh, gait monitoring

## Abstract

Gait event detection is essential for controlling an orthosis and assessing the patient’s gait. In this study, patients wearing an electromechanical (EM) knee–ankle–foot orthosis (KAFO) with a single IMU embedded in the thigh were subjected to gait event detection. The algorithm detected four essential gait events (initial contact (IC), toe off (TO), opposite initial contact (OIC), and opposite toe off (OTO)) and determined important temporal gait parameters such as stance/swing time, symmetry, and single/double limb support. These gait events were evaluated through gait experiments using four force plates on healthy adults and a hemiplegic patient who wore a one-way clutch KAFO and a pneumatic cylinder KAFO. Results showed that the smallest error in gait event detection was found at IC, and the largest error rate was observed at opposite toe off (OTO) with an error rate of −2.8 ± 1.5% in the patient group. Errors in OTO detection resulted in the largest error in determining the single limb support of the patient with an error of 5.0 ± 1.5%. The present study would be beneficial for the real-time continuous monitoring of gait events and temporal gait parameters for persons with an EM KAFO.

## 1. Introduction

KAFOs are prescribed for patients with weakness or paralysis of the quadriceps. There are two different KAFOs: those with a knee-locking device such as a ring, bail, dial, or plunger lock, and those that provide foot clearance in swing and thus allow knee flexion [1,2]. Those with a knee-locking device ensure knee stability during walking but do not allow knee flexion, resulting in compensatory behaviors such as hip hiking, contralateral vaulting, and circumduction gait and thus reducing the KAFO’s energy efficiency [2,3,4,5]. On the other hand, a KAFO without any knee-locking device provides free knee flexion and limits gait stability. 

EM KAFOs satisfy both the stability in stance and the foot clearance in swing [2,6,7,8,9]. Force sensitive resistor (FSR) sensors embedded in EM KAFOs have been used to detect gait events, especially initial contact (IC) and toe off (TO), which provide contact information for the KAFO control to determine the appropriate knee flexion during gait. De Miguel-Fernández et al. [9] reviewed control strategies in studies using lower limb exoskeletons and reported that the measurement of vertical ground reaction forces (GRFs) was the most common in determining temporal gait events. Irby et al. [1,8] and Hwang et al. [2] used FSR sensors to control a wrap spring clutch, allowing knee flexion during swing. These studies showed that EM KAFOs (or stance control KAFOs) allowed less energy consumption than the locked KAFOs. FSR sensors accurately detect gait events but lack mechanical robustness and durability [10,11]. Instead, inertial measurement units (IMUs), which contain an accelerometer and a gyroscope and are easy to use, small, and lightweight, are used to detect gait events [10,12]. Piriyakulkit et al. [12] conducted a study to detect IC and TO using IMU sensor signals from the thigh position to develop an assistive device for lumbar kyphosis. They detected IC and TO with an average error rate of −33.6 ms and 38.7 ms at four different speeds (1, 2, 3, and 4 kph). Maqbool et al. [10] used an IMU sensor attached to the shank to detect IC and TO in the development of a below-knee prosthesis, and the detection errors during level walking were 10.7 ± 17.9 ms and −7.6 ± 35 ms for IC and TO, respectively.

Gait event detection is essential not only for controlling KAFOs [13] but also for determining gait parameters. These gait parameters are clinically essential and useful tools for assessing various pathologies in patients and quantifying rehabilitation outcomes [14,15,16]. Spatiotemporal gait parameters evaluate the patients’ gait and indicate disease progression or improvement [16,17]. The parameters include stride time, step time, stance, swing, cadence, gait symmetry, double limb support, and single limb support. These can be calculated only with four essential gait events: initial contact (IC), toe off (TO), opposite initial contact (OIC), and opposite toe off (OTO).

It is important to determine the essential gait parameters of patients after the prescription of an orthosis since they serve as a representation of physiotherapy assessment and training progress [18,19]. Hebert et al. [18] emphasized the importance of gait training for sequencing the relearning and achieving optimal ambulation after the prescription, suggesting the real-time monitoring of a patient’s gait pattern with any portable device. Similarly, Andreoni et al. [19] focused on orthotic evaluation protocols based on gait analysis. They attached an accelerometer on the patients’ trunk to detect IC during gait in post-polio patients and determined step parameters such as stride/step time and cadence. However, they did not report other important temporal gait parameters such as symmetry and single/double limb support, since only the IC was measured.

Schlachetzki et al. [16] used IMU sensors attached to both sides of shoes to detect the IC and TO, calculating the stance and swing time for gait parameters in patients with Parkinson’s disease. Ledoux [17] used angular velocity data from the shank to detect the IC and TO with error rates of −1.7 ± 0.6% and −1.8 ± 0.6%, respectively, based on a threshold method. Gurchiek et al. [20] also detected the IC and TO via zero crossing of filtered acceleration signals measured at the thigh, with errors of 39 ± 28 ms and 28 ± 28 ms, respectively. In general, up to now, studies with IMU sensors placed on the unilateral side have determined only the IC and TO. Therefore, essential gait parameters such as gait symmetry and single/double limb support could not be determined. González et al. [21] used vertical and anteroposterior accelerations at the L3 vertebra of young and healthy subjects and detected the IC and TO with errors of 13 ± 35 ms and 9 ± 54 ms, respectively. McCamley et al. [22] also attached an IMU sensor on the lower lumbar spine and detected the IC and TO by smoothing and differentiating the vertical acceleration signal with a Gaussian CWT. Their results reported that the IC and TO were detected with an error rate of 2% and 3%, respectively.

In this study, we developed a gait event detection algorithm to compute temporal gait parameters from patients with an EM KAFO. Two key aspects were considered: utilizing a single IMU sensor embedded in the KAFO and detecting four major gait events (IC, TO, OIC, and OTO). Previous studies have primarily focused on attaching a single IMU sensor to the center of mass (COM), thigh, or shank for gait event detection [10,12,17,20,21,22,23]. The thigh and shank, integral components of a KAFO, are commonly chosen as locations for sensor attachment and serve as primary sites for module attachment for control purposes. However, studies employing an IMU sensor on thigh or shank [10,12,17,20,23] were limited to detecting only the IC and TO. This limitation poses a constraint on identifying all four gait events, which, in turn, requires sensor attachment on both legs. Additionally, while it is possible to obtain contact information for both feet when the sensor is positioned at the COM, it is not an appropriate location for considering the use of a sensor embedded in the KAFO. Hence, in this study, we developed an algorithm to detect four gait events (IC, TO, OIC, and OTO) to determine essential temporal gait parameters using a single IMU sensor embedded in the thigh part of an EM KAFO. The threshold-based algorithm was developed, and its evaluation was performed on two healthy adults and a hemiplegic patient wearing an EM KAFO.

## 2. Materials and Methods

### 2.1. Gait Event Detection Algorithm

**Figure 1:**
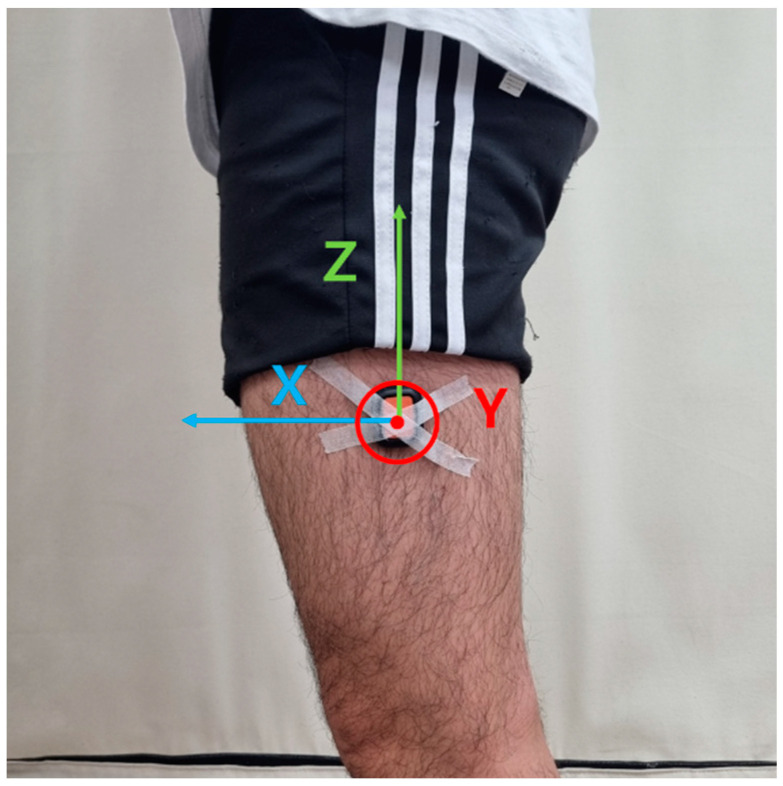
The IMU sensor orientation. X axis: anterior–posterior direction, Y axis: medial–lateral direction, and Z axis: superior–inferior direction.

Figure 1 shows the IMU sensor orientation for this study. The X, Y, and Z axes are the anterior–posterior (AP), the medial–lateral (ML), and the vertical (V) direction, respectively. Table 1 represents the signals used in the algorithm. The X axis and Z axis accelerations (AccX and AccZ) and the pitch angular velocity signal (GyroY) were selected as feature signals. The AccZ was filtered with a second-order Butterworth band-pass filter with the main frequency ± 0.5 Hz (AZbp) [24]. The AZbp showed two sine waveforms in a single gait cycle. The GyroY was low-passed using a second-order Butterworth filter with a 3 Hz cutoff frequency (GYlp3).

**Figure 2:**
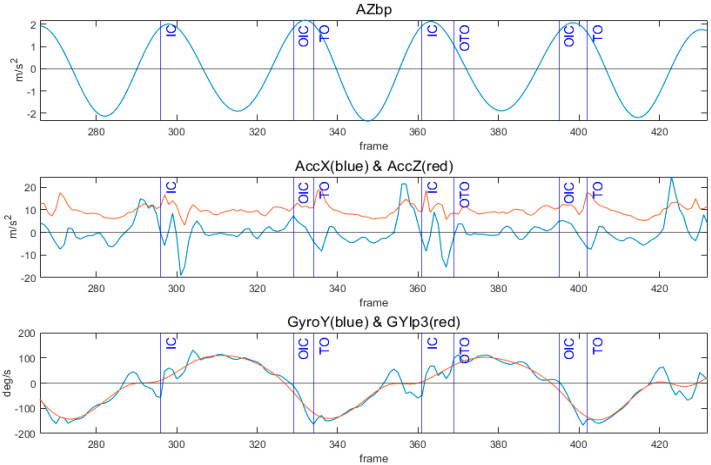
Signals used in gait event detection (AZbp, AccX, AccZ, GyroY, and GYlp3) with reference points from four force plates. Blue and red lines in the second plot represent AccX and AccZ, respectively. Similarly, blue and red lines in the third plot represent GyroY and GYlp3, respectively.

**Figure 3:**
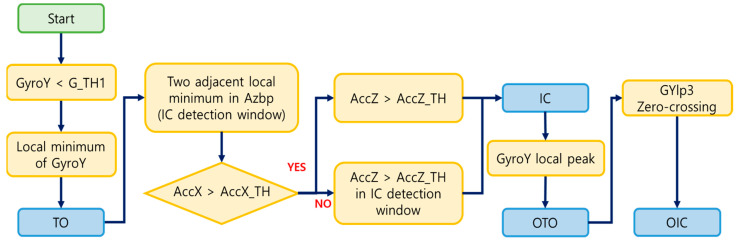
Flow chart of the gait event detection algorithm.

Figure 2 illustrates an example of the feature signals and detection of four gait events (IC, TO, OIC, and OTO). To identify TO, the local minimum point of GyroY was determined, occurring when GyroY becomes smaller than a specific threshold [12]. This threshold was set to point out TO among many local minima, and its value was determined through a grid search, ensuring precision in event detection. In the detection of IC, indicating when the foot first contacts the ground, the algorithm found the peak in the acceleration signal, implying an impact. The IC detection window, defined between two adjacent local minima of the AZbp, allowed stable peak point detection in acceleration. Thresholds were set for the AccX and AccZ to identify the most prominent peaks in each signal, and these thresholds were fine-tuned through a grid search methodology. The AccX above the threshold was first checked within the detection window. If the AccX satisfied this condition, a peak above the threshold of the AccZ was defined as the IC. If not, the IC was the peak above the threshold in the AccZ within the IC detection window. The detection of OTO occurred as the local peak in GyroY after IC, with a set time delay ensuring identification after a specific interval in the gait cycle compared to IC. Finally, after OTO, OIC was defined as the point at which GYlp3 zero crossed from (+) to (−). Figure 3 presents the flow chart of the algorithm.

### 2.2. Validation of the Algorithm Using Data with EM KAFO

**Figure 4:**
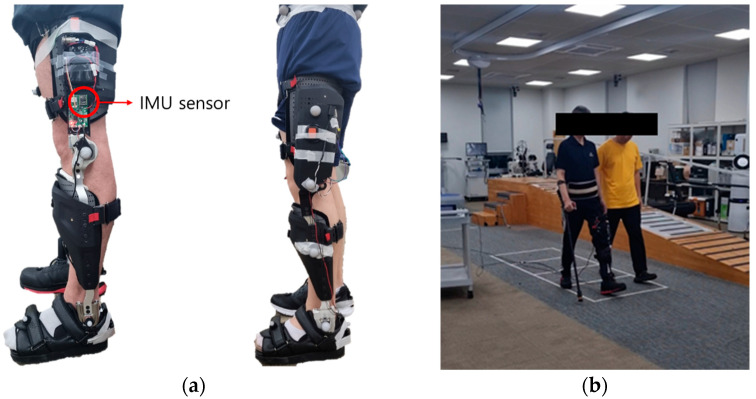
(**a**) Two types of EM KAFOs (left: one-way clutch KAFO, right: pneumatic cylinder KAFO); (**b**) gait experiment for a patient with an EM KAFO.

Gait experiments for persons with an EM KAFO were conducted to evaluate the developed algorithm. Two different types of EM KAFOs were developed by the Korea Orthopedics and Rehabilitation Engineering Center (KOREC): a one-way clutch KAFO and a pneumatic cylinder KAFO (Figure 4). The one-way clutch KAFO was designed to prevent unexpected flexion of the knee joint during the stance phase. On the other hand, the pneumatic cylinder KAFO adjusted the resistance of the knee joint based on the sensor values during the gait cycle.

The KAFO-worn experiment included two healthy adult males (38.5 ± 4.9 years, 179.5 ± 4.9 cm, 76.0 ± 1.4 kg) and one patient (61 years, 173 cm, 62 kg) with left hemiplegia. The patient was diagnosed with an incomplete SCI due to an accident in 2015 and was primarily capable of walking independently using a cane, with an mFAC score of 4 to 5. Informed consent was obtained from all subjects prior to their participation in this study. The experiment received an approval from the institutional review board in the Korea Orthopedics and Rehabilitation Engineering Center (RERI-IRB-221208).

The IMU (EBIMU-9DOFV5; E2BOX, Seoul, Korea) embedded in the KAFO was positioned at 18 cm superior to the knee joint and measured three-axis acceleration and three-axis angular velocity signals at 100 Hz. The reference gait events were measured with a sampling frequency of 1 kHz using four force plates (AMTI, Watertown, MA, USA) in the gait laboratory. The number of strides measured in the experiment is shown in Table 2.

Frequency analysis was performed to confirm the main characteristics of the EM KAFO-worn gait data, and the results are shown in Table 3. The main frequency of the Z axis acceleration was 1.6 Hz for the healthy adults and 1.4 Hz for the patient. Based on these results, the filtering bandwidth of the AZbp signal was determined [24].

### 2.3. Evaluation of Gait Event Detection Algorithm

The evaluation of the algorithm was carried out by calculating the error and the error rate (Equations (1) and (2)). The error was calculated as the time difference between the detected point and the reference point. The error rate was expressed as a percentage by dividing the error by the duration of a gait cycle.

Error (ms) = Detected − Reference (1)

Error rate (%) = (Error / 1 cycle duration) × 100%(2)

In addition, the essential gait parameters such as the stance, swing, and single/double limb support periods were determined and compared with those measured for the reference gait events. The symmetry index was defined by the percentage of time from the IC to the OIC with respect to one gait cycle.

## 3. Results

**Figure 5:**
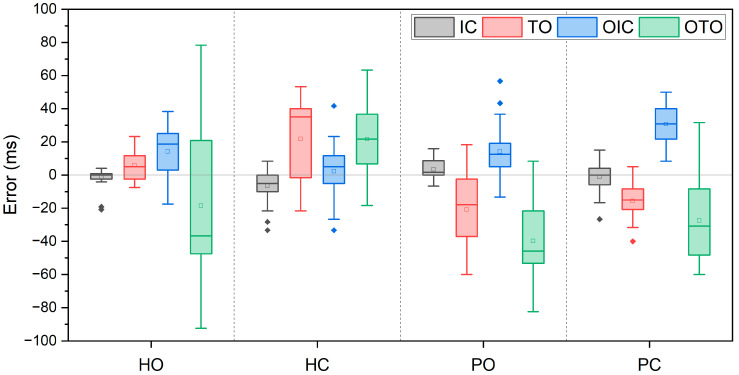
Mean errors (ms) in detection of four gait events compared with the refence. HO: healthy adult with one-way clutch KAFO, HC: healthy adult with pneumatic cylinder KAFO, PO: patient with one-way clutch KAFO, and PC: patient with pneumatic cylinder KAFO. ◆ represents outliers of the box plot that exceed 3 times the IQR.

Errors between the detected and the reference gait events are shown as box plots in Figure 5. The diamond symbols (◆) within the box plot indicate outliers, defined as values exceeding three times the interquartile range (IQR). Denoted as HO, HC, PO, and PC, these labels represent healthy adults with a one-way clutch KAFO, healthy adults with a pneumatic cylinder KAFO, patients with a one-way clutch KAFO, and patients with a pneumatic cylinder KAFO, respectively. Among four gait events, IC (black) showed the smallest error and variation for all cases (HO: −1.2 ± 5.4 ms, HC: −6.5 ± 10.5 ms, PO: 3.4 ± 5.2 ms, PC: −1.2 ± 7.7 ms). TO (red) exhibited the largest variation for HC (21.8 ± 24.6 ms), and PO followed (−20.8 ± 22.3 ms). For PC, the largest error occurred in OIC (blue), measuring 30.7 ± 11.8 ms. The largest error was observed in OTO (green) for PO (−39.9 ± 22.0 ms), and the largest variation was in the OTO for HO (30.7 ± 11.8 ms).

Table 4 shows the results of gait event detection in healthy adults and a patient with an EM KAFO. The ‘−’ sign indicates early detection of the event. Overall, the error rates were calculated to be within 3% for all cases. In healthy adults, the smallest error rate was observed in IC for HO (−0.1 ± 0.5%) and OIC for HC (0.2 ± 1.3%). Conversely, the first and the second largest error rates in HC were shown for TO and OTO with 1.8 ± 2.0% and 1.8 ± 1.9%, respectively.

For the patient, regardless of the KAFO type, OTO showed the largest error rate among all events (PO: −2.8 ± 1.5%, PC: −2.5 ± 1.4%). Conversely, the smallest error rate was in IC for both KAFO types (PO: 0.2 ± 0.4%, PC: −0.1 ± 0.6%). The error rate in detecting OIC for PC was larger than that for PO.

Temporal gait parameters for HO, HC, PO, and PC are shown in Table 5. ‘Measured’ corresponds to the reference gait parameters from force plates, and ‘Calculated’ is the value determined by the detected events. The difference between ‘Calculated’ and ‘Measured’ is presented as ‘Error’ (Error = Calculated – Measured). In HC, single limb support was determined with the smallest error (−0.9 ± 2.1%), and stance time showed the largest error (2.7 ± 1.6%). Conversely, in HO, the smallest error occurred in stance time (0.7 ± 0.8%), while the largest error was observed in single limb support (3.8 ± 3.4%). The symmetry index for PO showed the smallest error (0.9 ± 1.0%), closely followed by the stance time (−1.3 ± 1.1%). In PC, the stance time had the smallest error among the gait parameters (−0.9 ± 0.6%). Moreover, the symmetry index for PC exhibited an error of 2.5 ± 0.8%, which was larger than that in PO. For both KAFO types, single limb support showed the largest error (PO: 3.5 ± 2.4%, PC: 5.0 ± 1.5%), and the second largest error was determined in double limb support (PO: −2.5 ± 1.6%, PC: −2.7 ± 1.2%).

## 4. Discussion

In this study, an algorithm was developed to monitor the temporal gait parameters of patients with an EM KAFO based on four key gait events (IC, OTO, OIC, and TO). It is crucial to measure the temporal gait parameters of patients wearing KAFOs to assess their walking condition, enable precise corrections, and implement interventions in orthoses. Furthermore, acquiring information about the four key gait events enables the computation of detailed gait parameters such as stance, swing, symmetry, and single/double limb support, amplifying the significance of the overall assessment process. The algorithm was evaluated by conducting gait experiments on healthy adults and a hemiplegic patient with two different prototypes of EM KAFOs.

As shown in Table 4, the error rates for IC, OTO, OIC, and TO in the two healthy adults (HO, HC) and the patient (PO, PC) were below 2% and 3%, respectively. IC was most accurately detected by the developed algorithm, while OTO exhibited the largest error rate for all cases. Furthermore, for OTO, the patient (PO, PC) showed error rates of −2.8 ± 1.5% and −2.5 ± 1.4%, respectively. The IMU sensor in this study had a sampling rate of 100Hz, and one gait cycle for the patient was approximately 1.4 s. With an error rate of 1–2%, representing an error within three frames, our results indicate a relatively small error and a good performance. Additionally, while some differences were observed between the patient and the healthy adults, the results were either similar to or better than those reported in previous studies [17,20,22,23,25]. Table 6 represents the comparison of results with other studies.

The IMU sensor was attached to the COM for gait event detection in previous studies [21,22]. The COM would be an appropriate location for a single IMU sensor since IC and TO for both legs could be determined. McCamley et al. [22] determined IC and TO with an error rate of 2% and 3%, respectively, by attaching an IMU sensor to the COM. The error rates for IC and TO in this study were smaller than those reported in the previous study, indicating the better performance of the present algorithm. Additionally, the COM was not considered for the sensor location in this study, as the IMU sensor embedded in the developed EM KAFO was the only one needed.

Some studies used an IMU sensor attached to the lower limb. Ledoux [17] determined the IC and TO using angular velocity signals from the shank with error rates of −1.7 ± 0.6% and −1.8 ± 0.6%, respectively. Gurchiek et al. [20] used the IMU signal from the thigh and determined the IC and TO with errors of 39 ± 28 ms and 28 ± 28 ms, respectively. In the present study, mean errors were found to be −3.5 ± 8.3 ms and 12.7 ± 19.0 ms for IC and TO in healthy adults, respectively, which is relatively no different to the two previous studies. However, it is noted that most previous studies with an IMU sensor on the unilateral side of the limb could detect only the IC and TO. On the contrary, our study successfully detected four gait events with a single IMU on the lower limb.

Ding et al. [25] attached an IMU sensor on the dorsal surface of a shoe to detect four key gait events such as IC, TO, FF (foot-flat), and HO (heel-off). They reported that the detection error for each event was −10 ms, 21 ms, 19 ms, and 40 ms, respectively. Their results showed no difference to our results, considering the sample rates of the measurement. Garcia et al. [23] also determined IC, TO, FF, and HO using an IMU sensor in the thigh pocket with the hidden Markov model and reported detection errors of 36.7 ms, 6.7 ms, 23.3 ms, and −6.7 ms, respectively. That study attached the sensor in a similar location to the one used in our study and detected four gait events; however, a distinction lies in the use of machine learning methods in their research.

Including symmetry index, a crucial aspect in simple gait analysis [26,27], gait parameters were successfully determined in this study by identifying the IC, TO, OIC, and OTO. While the stance time errors for PO and PC were small at −1.3 ± 1.1% and −0.9 ± 0.6%, respectively, comparing PO and PC, the symmetry index for PO was more accurate than that for PC, possibly influenced by the 2.1% detection error in OIC for PC (Table 3). Early detection of OTO seemed to affect the error in single/double limb supports, leading to a longer single limb support and a shorter double limb support.

For PO, the same gait experiment was repeated three times (week 3, week 10, and week 11) and showed that the main frequency of the acceleration increased gradually. On the other hand, the main frequency for healthy adults was maintained during the two-day experiments (week 3 and week 10). This suggested that the gait improvement was made over time by practicing and as the patient got used to the KAFO.

Recently, many researchers have used machine learning (ML) or deep learning (DL) models such as LSTM [28,29], CNN [30], and random forest [31] to determine gait events or to classify gait phases. It was difficult to directly compare the results from the machine learning method with the present study. However, ML- or DL-based algorithms require more computation time to extract important features and to train the model [10,32]. Therefore, in this study, a threshold-based method was used to monitor gait events or parameters. However, considering the efficacy of machine learning approaches in yielding precise results, it appears worthwhile to compare them with our threshold-based methods in future studies.

Limitations of the present study include a lack of subjects who participated in the gait experiment with EM KAFOs. It was hard to recruit patients wearing EM KAFOs and difficult to fit the KAFO to them. Although only one hemiplegic patient was tested in this study, no significant difference in the error rates of gait event detection was found between the patient and the two healthy subjects. Further monitoring studies with additional patients will be performed in the future.

## 5. Conclusions

In this study, a gait event detection algorithm was developed to determine gait parameters for patients with an EM KAFO using an embedded IMU sensor on the thigh. This algorithm could detect four main gait events (IC, OTO, OIC, and TO) surpassing the capabilities of previous research limited to the identification of only two events using a single IMU attached to the lower limb. The determination of crucial parameters such as stance/swing time, symmetry, and single/double limb supports is vital for assessing the progression of diseases or rehabilitation in patients. The gait experiments involving two healthy adults and a hemiplegic patient, all wearing an EM KAFO, yielded promising results. For all cases, the error rates were less than 3% compared to the reference values. Notably, our algorithm demonstrated the smallest error rate in IC detection, while OTO exhibited the largest, which resulted in a most significant error of only 5% in determining single/double limb support. The potential impact of this research lies in the application of our algorithm, expected to be embedded in an application for real-life monitoring of gait parameters in KAFO-wearing patients. Our study contributes to the improvement of patient care and rehabilitation strategies.

## Figures and Tables

**Table 1:** Feature signals used in the gait event detection.

Feature Signal	Description
AccX	X-axis acceleration signal (AP direction)
AccZ	Z-axis acceleration signal (V direction)
AZbp	The signal filtered with a bandpass filter on AccZ (cutoff frequency = (main frequency of AccZ ± 0.5 Hz))
GyroY	Angular velocity in the pitch direction
GYlp3	The signal filtered with a bandpass filter on AccZ (cutoff frequency = 3 Hz)

**Table 2:** The number of strides collected from the experiments.

	Type of KAFO	Strides
Healthy adults	One-way clutch	20
	Pneumatic cylinder	15
Patient	One-way clutch	22
	Pneumatic cylinder	12

**Table 3:** Main frequency of AccX, AccY, and AccZ. Frequencies are shown as mean ± SD (standard deviation).

Acceleration	Main Frequency (Hz)	
	Patient	Healthy Adults
AccX	1.4 ± 0.1	1.6 ± 0.1
AccY	0.7 ± 0.1	0.8 ± 0.1
AccZ	1.4 ± 0.1	1.6 ± 0.1

**Table 4:** Error rates (mean ± SD) of the gait event detection for the EM KAFO-worn gait.

Error Rate (%GC)	IC	TO	OIC	OTO
HO	−0.1 ± 0.5	0.5 ± 0.7	1.1 ± 1.3	−1.3 ± 3.9
HC	−0.5 ± 0.8	1.8 ± 2.0	0.2 ± 1.3	1.8 ± 1.9
PO	0.2 ± 0.4	−1.5 ± 1.5	1.0 ± 1.0	−2.8 ± 1.5
PC	−0.1 ± 0.6	−1.2 ± 0.8	2.1 ± 0.8	−2.5 ± 1.4

**Table 5:** Comparison of gait parameters between algorithm-based and reference measurements.

Mean ± SD (%)		Stance	Symmetry	Single Limb Support	Double Limb Support
HO	Measured	62.0 ± 1.5	48.2 ± 1.4	33.3 ± 1.6	14.1 ± 1.1
	Calculated	62.8 ± 1.3	49.6 ± 1.3	37.1 ± 3.9	12.5 ± 1.4
	Error	0.7 ± 0.8	1.3 ± 1.4	3.8 ± 3.4	−1.2 ± 2.1
HC	Measured	62.2 ± 1.3	48.9 ± 1.4	34.5 ± 1.2	13.4 ± 1.2
	Calculated	64.9 ± 1.1	49.9 ± 1.4	33.6 ± 1.9	15.4 ± 1.7
	Error	2.7 ± 1.6	1.0 ± 1.5	−0.9 ± 2.1	1.9 ± 1.9
PO	Measured	63.0 ± 2.4	49.2 ± 2.7	34.2 ± 2.3	14.9 ± 1.4
	Calculated	61.6 ± 3.2	50.0 ± 3.3	37.7 ± 3.4	12.3 ± 1.6
	Error	−1.3 ± 1.1	0.9 ± 1.0	3.5 ± 2.4	−2.5 ± 1.6
PC	Measured	62.3 ± 1.1	47.3 ± 1.1	33.1 ± 0.9	15.1 ± 0.9
	Calculated	61.4 ± 1.0	49.7 ± 1.4	38.1 ± 1.7	12.3 ± 0.7
	Error	−0.9 ± 0.6	2.5 ± 0.8	5.0 ± 1.5	−2.7 ± 1.2

**Table 6:** Comparison with other studies.

Feature Signal	This Study	McCamley et al. [22]	Ledoux [17]	Gurchiek et al. [20]	Ding et al. [25]	Gracia et al. [23]
Subjects	2 healthy, 1 hemiplegic	18 healthy	10 healthy, 5 TFAs *	32 healthy	10 healthy	9 healthy
Sensor (position)	IMU (thigh)	IMU (COM)	IMU (shank)	IMU (thigh)	IMU (foot)	IMU (thigh)
Detected events	IC, TO, OIC, OTO	IC, TO	IC, TO	IC, TO	IC, TO, FF, HO	IC, TO, FF, HO
Method	Threshold (Acc, Gyro)	Threshold (Acc)	Threshold (Gyro)	Threshold (Acc)	Threshold (Acc, Gyro)	Hidden Markov Model
Performance (error rate, %)	IC: −0.1 ± 0.6 TO: −0.2 ± 1.9 OIC: 1.0 ± 1.3 OTO: −1.1 ± 2.9	IC: 2 TO: 3	IC: −1.7 ± 0.6 TO: −1.8 ± 0.6	**–**	**–**	**–**
Performance (error, ms)	IC: −1 ± 8 TO: −3 ± 24 OIC: 15 ± 17 OTO: −18 ± 38	IC: −6 ± 24 TO: −29 ± 26	**–**	IC: 39 ± 28 TO: 28 ± 28	IC: -10 TO: 21 FF: 19 HO: 40	IC: 36.7 TO: 6.7 FF: 23.3 HO: −6.7

* TFAs: transfemoral amputees.

## Data Availability

The data presented in this study are available upon request from the corresponding author. The data are not publicly available because the authors are continuing the study.
